# Predictors of Arterial Stiffness in Law Enforcement Officers

**DOI:** 10.3390/ijerph181910190

**Published:** 2021-09-28

**Authors:** Jason M. Keeler, Bradley S. Fleenor, Jody L. Clasey, Arnold Stromberg, Mark G. Abel

**Affiliations:** 1 Department of Kinesiology and Health Promotion, University of Kentucky, Lexington, KY 40506, USA; bsfleenor@bsu.edu (B.S.F.); jlclas0@uky.edu (J.L.C.); mark.abel@uky.edu (M.G.A.); 2 Department of Kinesiology, School of Public Health, Indiana University, Bloomington, IN 47401, USA; 3 Clinical Exercise Physiology, Human Performance Laboratory, Ball State University, Muncie, IN 47306, USA; 4 Department of Statistics, University of Kentucky, Lexington, KY 40506, USA; astro11@email.uky.edu

**Keywords:** carotid-femoral pulse wave velocity (cfPWV), cardiovascular disease (CVD), law enforcement officers (LEOs), arterial stiffness

## Abstract

Background: Compare arterial stiffness among law enforcement officers (LEOs) versus general population normative values and identify predictors of arterial stiffness in LEOs. Methods: Seventy male LEOs (age: 24–54 years) completed body composition, blood pressures, physical activity level, and carotid-femoral pulse wave velocity (cfPWV) measurements. T-tests and regression analyses were utilized to compare LEO data to normative data and predict cfPWV, respectively. Results: Compared to similar age strata within the general population, cfPWV was lower among LEO’s under 30-years (mean difference = −0.6 m·s^−1^), but higher among LEOs 50–55-years (mean difference = 1.1 m·s^−1^). Utilizing regression, age, relative body fat, and diastolic blood pressure explained the greatest variance in LEO’s cfPWV (adj. R^2^ = 0.56, *p* < 0.001). Conclusion: This investigation demonstrated that arterial stiffness may progress more rapidly in LEOs and LEOs’ relative body fat and blood pressure may primarily affect arterial stiffness and risk of CVD.

## 1. Introduction

Cardiovascular disease (CVD) is the leading cause of death in the United States [1]. The development of CVD occurs over a lifetime and is influenced by a variety of behavioral factors such as smoking, poor nutrition, physical inactivity, stress management, and others [1,2,3]. Law enforcement populations (police officers, special weapons and tactics units, sheriffs, etc.) are particularly susceptible to CVD. The prevalence of CVD among active duty law enforcement officers (LEOs) has been suggested to be like the general population [4]. However, CVD prevalence doubles among LEOs following retirement compared to the general population [5,6]. The rapid progression of CVD in LEOs necessitates the investigation of CVD risk factors and assessments. Earlier detection and intervention may delay the onset of CVD and/or reduce the occurrence of CVD events among active and retired LEOs.

Investigators have studied the prevalence of several traditional CVD risk factors in LEOs and found higher rates of hypertension and obesity in LEOs compared to the general population [4,5,6,7]. In addition, many LEOs lack the necessary physical activity to maintain cardiovascular health [3,4,5], while also experiencing intermittent periods of high physical and psychological stress, some of which are associated with performing law enforcement shift work [8,9]. Chronic stress is correlated with an increased risk of CVD [7,8]. Due to the deleterious health consequences and health care costs associated with CVD, the United States Centers for Disease Control (CDC) and National Institute for Occupational Safety and Health (NIOSH) created a goal to decrease CVD among public safety occupation including law enforcement [10]. The primary goals of the CDC’s agenda focused on conducting etiological studies of occupational risk factors and identification of work organizational factors that are associated with higher CVD risk in LEOs.

Several investigations have described a variety of occupational risk factors and stressors that LEOs are exposed to regularly, which include sudden onset and chronic exposure of physical and psychological stressors [2,3,4,7,11]. Psychological stress has been associated with vascular dysfunction, CVD, impaired sleep, and depression [9]. Likewise, Varvarigou et al. demonstrated that physical stress, such as performing physically demanding occupational tasks (e.g., restraints/altercations, pursuits of suspects, and physical training), was associated with sudden cardiac death, by serving as a physiological trigger to those who are predisposed to CVD [12]. Research in other tactical populations has noted that arterial stiffness increases during prolonged performance of occupational tasks [13]. In fact, arterial stiffness, an independent predictor of cardiac events, is associated with sudden cardiac deaths among firefighters [13]. Collectively, these data provide some insight into the cardiovascular health of LEOs, however utilization of direct measurements of cardiovascular health, such as arterial stiffness, would provide a clearer understanding of why LEOs are disproportionately at risk of CVD. In turn, appropriate interventions may be identified and employed, at earlier time points.

The measurement of arterial stiffness provides practitioners with an objective assessment that is predictive of CVD events [14,15,16,17,18]. Arterial stiffness, as measured by the gold-standard of carotid-femoral pulse wave velocity (cfPWV), has been found to independently predict cardiovascular events and all-cause mortality in numerous epidemiological studies [14,15,16,17,18]. The assessment of central arterial stiffness can detect earlier changes in the vasculature compared to brachial (i.e., peripheral) blood pressure measurements [16]. Central stiffness measurements, as measured by cfPWV, have been found to be superior in predicting first cardiovascular events compared to typical brachial blood pressure measurements [18]. Normal aging causes progressive escalation of arterial stiffness and hypertension [19]; however, excess body fat and a sedentary lifestyle further augment the increase in arterial stiffness and blood pressure [20]. To date arterial stiffness has not been assessed in LEOs, which may provide unique insight for increased CVD risk in LEOs.

Identifying lifestyle, occupational, and demographic factors that are associated with arterial stiffness will provide healthcare clinicians with an understanding of how to identify LEOs who may be at greater risk for CVD. Furthermore, identification of risk factors will provide practitioners and police departments with information to develop appropriate interventions to reduce CVD risk. Therefore, the purposes of this study were to compare the arterial stiffness of LEOs versus the general population and to identify lifestyle, occupational, and demographic predictors of arterial stiffness in LEOs. It was hypothesized that cfPWV would be greater in LEOs than in the general population (relative to age and gender) and cfPWV would be positively correlated to age, relative fat mass, years spent on third (night) shift, perceived stress, and inversely correlated to daily time spent in moderate-to-vigorous physical activity.

## 2. Materials and Methods

### 2.1. Experimental Design

This study utilized a cross sectional design to compare arterial stiffness in LEOs versus the general population and to identify predictors of arterial stiffness in a cohort of professional LEOs. Regarding the comparison of arterial stiffness in LEOs versus the general population, the subjects’ occupation (i.e., LEO vs. non-LEO) served as the independent variable and cfPWV served as the dependent variable. Regarding the prediction of arterial stiffness, age, body composition, shift outcomes, perceived stress, and physical activity outcomes served as predictor variables and cfPWV served as the dependent variable. The study was approved by the University’s Institutional Review Board (IRB) prior to subject recruitment and data collection.

### 2.2. Subjects

A sample of 70 professional LEOs recruited from the state of Kentucky and southwest Ohio participated in this study. Subjects were male professional LEOs between 21 and 55 years of age. Subjects were free of CVD according to the American College of Sports Medicine Guidelines [21]. Subjects were excluded from the study if they were a current smoker or reported any sign, symptom, or diagnosis of cardiovascular, pulmonary, or metabolic disease, because cfPWV changes have been noted in these individuals [14,22,23]. Subjects provided written informed consent prior to participation in the study and were informed that participation in the study would not affect their employment status or union membership.

### 2.3. Procedures

Subjects participated in one testing session lasting approximately 2.5 h, located in the University’s Exercise Physiology Laboratory or an alternative IRB approved location (i.e., police department, station, or training facility). Subjects abstained from consuming caffeine and food for at least three hours prior to testing.

### 2.4. Questionnaires

Subjects completed a physical activity readiness questionnaire (PAR-Q), a general health history questionnaire, and a work/personal history questionnaire (i.e., identification of age, race/ethnicity, rank, job duties, years in military, living arrangement, amount of time spent on certain shifts, and physical activity questions). In the work history questionnaire subjects were asked to recall the number of months they spent on 1st, 2nd, and 3rd shift over the entirety of their career in an effort to identify time spent on 3rd shift. To assess occupational stress levels, subjects completed the Operational Police Stress Questionnaire (PSQ-Op; ICC = 0.92) and the Organizational Police Stress Questionnaire (PSQ-Org; ICC = 0.92) [24]. The PSQ-Op and PSQ-Org specifically assess the perceived stressors related to policing over the last 6 months, using a 7-point Likert-type scale. The PSQ questionnaires use the descriptive anchors of 1 (no stress at all) to 7 (a lot of stress).

### 2.5. Anthropometrics

Subjects were asked to wear athletic clothing for anthropometric assessments including a t-shirt and athletic shorts. Height was measured, without shoes (to the nearest 0.1 cm) using a portable stadiometer (HM200P, Charder Medical, Taichung City, Taiwan). Body mass was measured (to the nearest 0.1 kg) using an electronic scale (Health O Meter, Newell Brands, Hoboken, NJ, USA). Circumference measurements were taken (to the nearest 0.1 cm) at the waist, abdomen, and hip according to American College of Sports Medicine guidelines [21]. Specifically, circumference measurements were performed at the end of a normal exhalation and taken in rotational order. Each circumference was repeated until two trials were within 1 cm. Measurements were taken against the skin, except for the hip measurement, which was performed over athletic shorts.

### 2.6. Body Composition

Body composition was measured with a dual-frequency bioelectric impedance analyzer (BIA; Bodystat 1500; Bodystat Ltd., Isle of Man, UK). Subjects assumed a supine position on a non-conductive surface with the limbs slightly abducted from the body and hands placed in a pronated position. Surface sensor electrodes were placed on the subject’s right side according to manufacturer guidelines. A series of standardized low-level electrical currents (5 and 50 kHz) were released, and the voltage drop due to the impedance was detected across the sensor. The manufacturer’s proprietary prediction equation estimated the subjects’ body composition. The prediction equation estimates percent fat and has been validated against dual-energy X-ray absorptiometry (r = 0.88) [25].

### 2.7. Dietary Quality

Each subject completed a web-based National Institute of Health Dietary History Questionnaire, focused on recall of food frequency patterns over the previous year. Assessment of dietary quality was in interpreted in relation to the United States Dietary Guidelines and expressed in a 12-component composite Healthy Eating Index (HEI) score (Range: 0–100). Higher HEI scores represent superior dietary quality. Nutritional analyses were performed with a computer program (Diet*Calc Analysis Program, National Cancer Institute, Silver Spring, MD, USA), while the HEI component and composite scores were calculated on SAS 9.4 (SAS Institute, Cary, NC, USA). Twelve subjects were excluded from the dietary analysis due to failure to fully complete the questionnaire.

### 2.8. Physical Activity Monitoring

Daily physical activity outcomes were evaluated while on- and off-duty over one week with the use of a research grade triaxial accelerometer (GT3X, ActiGraph Inc., Pensacola, FL, USA). The accelerometer was worn on the waistband on the subjects’ right side at the midaxillary line during waking hours to provide physical activity data via step and activity counts. The activity count data were utilized to quantify volume, intensity, frequency, and duration of subjects’ physical activity. Freedson and coworkers’ [26] physical activity intensity (i.e., activity count) thresholds were applied to quantify time spent in sedentary, light, moderate, and vigorous intensity categories. The ActiGraph GT3X has been validated to quantify step counts and physical activity time, while also demonstrating acceptable inter-device reliability (r = 0.90–0.99) [27,28]. All data were downloaded and evaluated using the manufacturer’s software (ActiLife Version 6, ActiGraph, Pensacola, FL, USA). Additionally, subjects were asked to keep a written physical activity log to confirm the duration of these activities and provide a qualitative context.

Accelerometer data were sampled at 30 Hz and collapsed into 10-second epochs. Wear and non-wear time data were identified through ActiLife’s proprietary procedure via the Troiano algorithm [29]. Thirteen subjects were excluded from the physical activity analysis for not wearing the accelerometer for a minimum of 10 h on at least 4 days. Thus, 57 subjects’ data were utilized for physical activity analyses with an average wear time of 7.1 ± 1.4 days.

### 2.9. Pulse Wave Velocity Analysis

Carotid-femoral pulse wave velocity (cfPWV) is the criterion measurement of arterial stiffness and was assessed via transcutaneous tonometry of the carotid and femoral arteries with simultaneous ECG recording utilizing the SphygmoCor System (AtCor, Sydney, Australia) [18]. This measurement has been shown to be reliable within a tactical population (ICC = 0.88) [30]. The test-retest reliability of this measure in this sample was ICC = 0.84 (*n* = 70). The subjects assumed a supine position with each measurement performed on the right side of the body. The subject rested in the supine position for 10–15 min prior to the measurement. The carotid and femoral measurement sites were palpated to find the strongest pulse and identified with a washable marker. The linear distances from the carotid site to the sternal notch, to the navel, and then to the femoral site were measured with an inelastic tape measure and input into the SphygmoCor software (AtCor, Sydney, Australia).

Pulse wave analysis measurements were performed with transcutaneous tonometry at the radial artery, using the SphygmoCor System following manufacturer’s guidelines. Central aortic pulse pressures were estimated by validated transfer functions [14]. Pressure waveform measurements were only accepted if the operator index was greater than 80%, per manufacturer recommendations. Specifically, the operator index is a proprietary score in the SphymoCor System used to measure the reproducibility and strength of the radial pulse signal. In addition, heart rate variability was assessed during a five-minute sampling period using measures of Root Mean Square of Successive Differences (RMSSD) and standard deviation of normal to normal (SDNN) R-R intervals.

### 2.10. Statistical Analysis

Basic statistics (i.e., mean ± standard deviation) were used to describe demographic and outcome variables. One sample T-tests were conducted to determine if differences existed in pulse wave velocity values between LEOs and age-matched reference values from the general population [31]. The general population reference values were established through a European Collaboration from 8 countries and 16,867 subjects [31]. Pearson Product Moment correlations were used to assess relationships between independent variables and the dependent variables of central pressures and cfPWV. Furthermore, a one-way ANOVA was used to assess the main effect of age classification on cfPWV. Tukey HSD post-hoc analysis was used when main effects were identified. The normality of cfPWV outcomes within age strata was assessed using the Shapiro-Wilks test. Multivariate linear regression was utilized to identify significant predictors of cfPWV values within LEOs. Backward-stepwise regression analyses were performed on hypothesized variables (age, body fat, time on third shift, stress questionnaire scores, and time spent in moderate-to-vigorous physical activity) to provide the strongest predictor of cfPWV. Stepwise regression analyses were utilized to determine the predictability of cfPWV from all lifestyle, demographic, and occupational variables collected. For the regression analyses, the predicted values were plotted against the residual values. None of the described regression models produced visual patterns when predicted values were plotted against the residual values, indicating linear regression was an appropriate model for interpretation. Multicollinearity was controlled utilizing a variance inflation factor limit of ten. All analyses were performed using JMP^®^ (Version 11. SAS Institute Inc., Cary, NC, USA) statistical software. The level of significance for all statistical analyses were set at *p* < 0.05.

## 3. Results

A total of 70 male LEOs from seven law enforcement agencies participated in this investigation. Descriptive characteristics of the study’s sample are displayed in Table 1. The subjects’ mean BMI was classified as overweight and borderline obese according to a mean BMI of 29.4 ± 4.5 kg·m^−2^. Furthermore, the subjects’ mean relative body fat (i.e., 22.8 ± 5.6%) falls between the 20th and 30th percentile relative to age and gender [21]. The subjects’ mean HEI score (60.3 ± 12.6) was between the 75th and 90th percentile relative to the general population [21]. The subjects’ mean daily moderate-to-vigorous physical activity was 7.4 ± 11.0 min·d^−1^. Descriptive outcomes of resting cardiovascular measurements are presented in Table 2. Table 3 displays a comparison of mean PWV by age strata, between 70 male LEOs and normative values from the European Society of Cardiology. LEOs under 30 years of age had significantly lower average PWV than the normative value (Table 3; *p* = 0.0018). LEOs in the 4th and 5th decades of life demonstrated no significant differences compared to the normative values. However, the 50–55 year. group had a significantly higher PWV than the normative value (*p* = 0.0003).

Table 4 displays comparisons of descriptive and cardiovascular measures by age strata in 70 male law enforcement officers. Significant main effects across age strata were noted in age (F (3, 66) = 175.30, *p* < 0.0001), LEO experience (F (3, 66) = 34.01, *p* < 0.0001), relative body fat (F (3, 66) = 3.29, *p* = 0.0260), aortic augmentation index (F (3, 66) = 9.28, *p* < 0.0001), aortic augmentation index at a standardized heart rate of 75 b·min^−1^ (F (3, 66) = 10.11, *p* < 0.0001), and cfPWV (F (3, 66) = 17.86, *p* < 0.001) such that greater values were noted with increased age. Post-hoc analyses revealed that LEOs under the age of 30 years had significantly fewer years of experience and significantly lower relative body fat than the 50–55 years group (*p* = 0.0206). Post-hoc analysis also revealed that LEOs under the age of 30 years had significantly lower cfPWV compared to the 30–39 years group (*p* = 0.029), the 40–49 years group (*p* < 0.001), and the 50–55 years group (*p* < 0.001). The LEOs in the 6th decade of life had the highest mean cfPWV (9.4 m·s^−1^) and were significantly higher than all other groups (*p* < 0.0001). There was no effect of age on systolic or diastolic blood pressure across age strata.

Bivariate correlations were performed between arterial stiffness versus all demographic, anthropometric, lifestyle, and occupational variables. There was a significant positive correlation between age and cfPWV (r = 0.57, *p* < 0.01). This relationship suggests that as age increases cfPWV also increases. There were significant positive correlations between cfPWV versus years served in law enforcement (r = 0.60, *p* < 0.01) and relative body fat (r = 0.60, *p* < 0.01). These findings indicate that arterial stiffness increases with an increase of years served in law enforcement and increased body fat percentage. A significant positive correlation was also identified between the two predictor variables of years served in law enforcement and relative body fat (r = 0.34, *p* < 0.05). These findings suggest that there is an increase in percent body fat with an increase in years of service in law enforcement. Other significant positive relationships were also identified between cfPWV versus all other variables (see Supplementary Table S1, Bivariate correlations…) except the three police stress questionnaire variables (PSQ-op, r = 0.1; PSQ-org, r = −0.02; PSQ-total, r = 0.04).

Bivariate correlations demonstrated a moderate correlation between the predicted variable of time spent on 3rd shift and cfPWV (r = 0.31, *p* < 0.05). Therefore, utilizing the median value of 24 months on 3rd shift, subjects were stratified into two groups (less than two years on 3rd shift (*n* = 41) and two or more years on 3rd shift (*n* = 29)) to compare the group average of cfPWV. Although not significant, LEOs with 2 or more years of 3rd shift work had a slightly higher average cfPWV (6.94 ± 1.34 m/s^−2^) compared to those with less than 2 years of 3rd shift work (6.41± 1.35 m/s^−2^; *p* = 0.108).

Bivariate correlations demonstrated that two of the strongest correlates of cfPWV were relative body fat and age. Relative body fat and age were also correlated with each other. Thus, to describe the independent effects of relative body fat on cfPWV, a one-way ANCOVA was performed, with age serving as the covariate. Relative body fat was stratified into three groups (i.e., obese, overweight, and normal weight), according to normative data standards from the ACSM [21]. When controlling for age there was a main effect for body fat classification on cfPWV (Table 5). Post-hoc analyses revealed that the obese group had a greater cfPWV than the normal body weight group for relative body fat (F (3, 66) = 22.56, *p* < 0.001; mean difference = 1.46 m·s^−1^). Furthermore, the obese group had a greater cfPWV than the overweight group (F (3, 66) = 22.56, *p* = 0.001; mean difference = 0.45 m·s^−1^). Likewise, to describe the independent effects of body mass index on cfPWV, a one-way ANCOVA was performed, with age serving as the covariate (Table 5). There was a main effect for BMI. Post-hoc analyses revealed that the obese group had greater cfPWV than the normal BMI group for BMI (F (3, 66) = 16.38, *p* < 0.001; mean difference = 1.16 m·s^−1^).

Table 6 displays the three strongest multiple linear regression models used to predict cfPWV in male LEOs, with the occupational and lifestyle factors. As model A demonstrates, age and relative body fat explain 51% of the variance in cfPWV, and these are the two primary variables for most of the stronger regression models. Absolute fat-mass, and BMI were run in these models, but relative fat explained greater variance, and therefore it was utilized in the models. The addition of other hypothesized variables only slightly increased the explained variance for other models. Interestingly, the addition of the variable daily moderate-to-vigorous physical activity reduced the variance explained by the regression model. The addition of the non-hypothesized variable brachial diastolic blood pressure provided an increase of explained variance in Model B. Model C demonstrates the predictive power of using years in law enforcement compared to the hypothesized variable of Age in Table 6. There is slightly more explained variance using the variable years in law enforcement compared to the variable Age.

## 4. Discussion

Our hypothesis that cfPWV would be greater in LEOs than age-matched counterparts from the general population varied by age group. Interestingly, the <30 years old cohort had a significantly lower cfPWV compared to the age-matched reference group. There was no difference in the cfPWV between LEOs and the general population within 30–39 and 40–49 years old cohorts. However, the cfPWV of the ≥50 years old LEO cohort in the present study was 1.1 m·s^−1^ greater than the age-matched reference group (*p* < 0.001; Table 3). Our second hypothesis was that cfPWV would be positively correlated with age, fat mass, time spent on 3rd shift, perceived stress, and inversely correlated with daily time spent in moderate–to-vigorous physical activity. Carotid-femoral pulse wave velocity was positively correlated with age, fat mass, and time spent on third shift. Interestingly, there were no correlations between Police Stress Questionnaire and physical activity outcomes versus cfPWV in this investigation. Collectively, our findings indicate that LEOs may have lower cfPWV at the beginning of their careers but remolding of the arteries through the lifespan may accelerate causing arterial stiffening and increasing the risk of cardiovascular disease and events.

Despite the cross-sectional nature of this descriptive comparison, this apparent rise in arterial stiffness over the lifespan of LEOs is concerning, because of the increased risk of a cardiovascular event. A previous investigation’s meta-analysis demonstrated that an increase of 1 m·s^−1^ in cfPWV corresponded to an increased risk of 14% for all cardiovascular events, 15% increased risk for cardiovascular mortality, and a 15% increased risk of all-cause mortality [18]. Furthermore, it was reported that an increase of 1 standard deviation in cfPWV (i.e., cfPWV = 3.4 m·s^−1^) increased those risk outcomes to 47%, 47%, and 42%, respectively [18]. The Framingham Heart Study demonstrated that higher PWV values were associated with increased cardiovascular disease risk [17].

The <30 years LEO cohort’s lower cfPWV compared to the general population is intriguing and may be, in part, attributed to “the healthy worker effect.” The healthy worker effect occurs when unhealthy or potentially unhealthy workers are selectively omitted through demands imposed by occupational necessities. To become a LEO, recruits typically participate in several months of physical training in an academy to meet academy and/or department physical test requirements [5]. Indirectly, these requirements increase the health of the LEO cohort compared to the general population. Wu et al. demonstrated improved fitness and body composition outcomes in cadets following a 20-week training period [32]. Training academies may effectively reduce arterial stiffness, because resistance and endurance training in pre-hypertensive adults have been shown to reduce peripheral arterial stiffness, central blood pressures, and augmentation index [33]. On the other hand, the significantly higher cfPWV in the ≥50 years cohort begs the question of what is causing the acceleration in arterial stiffness beyond that of the general population over the 20-year career span.

Considering the apparent increase in arterial stiffness among LEOs over the career span, the second aim of this investigation was to identify demographic, lifestyle, and occupational predictors of arterial stiffness. It was hypothesized that cfPWV would be positively correlated with age, fat mass, time spent on 3rd shift, perceived stress, and inversely correlated with daily time spent in moderate–to-vigorous physical activity. Carotid-femoral pulse wave velocity was positively correlated with age, fat mass, and time spent on third shift. Interestingly, there were no correlations between Police Stress Questionnaire and physical activity outcomes versus cfPWV in this investigation. Regression analysis demonstrated that age, relative body fat, diastolic blood pressure, and years in law enforcement (Table 6) explained the most variance in cfPWV.

Aging has been established as a primary risk factor for CVD and is highly associated with increased arterial stiffness [18,19,34,35]. Several studies note a linear increase in cfPWV until the age of 60 years [16,17,34]. Following the age of 60 years, there tends to be a rapid exponential increase in cfPWV [16,17,34]. This increase is hypothesized to be due to the cyclical hemodynamic stresses that are placed on arterial structures, particularly elastin, which causes fracturing and improper remolding of the arterial matrix [15]. This occurs independently of atherosclerotic plaque accumulation; however, in some individuals atherosclerotic plaque accumulation may also be contributing factor to increased cfPWV. This cross-sectional investigation concurs with previous findings indicating that greater cfPWV values are associated with older LEOs (Table 3 and bivariate correlations). These results suggest that a function of time causes an unavoidable progression in arterial stiffening, however the degree to which this progression occurs could be modulated through other demographic, lifestyle, and occupational variables.

Given the extended period one spends in an occupation over a lifetime, factors associated with the occupation can have negative health consequences. The occupation of law enforcement has demonstrated a clear relationship of increasing the prevalence of traditional risk factors for negative health outcomes, like CVD, stroke, heart attack, post-traumatic stress syndrome, amongst others [2,3,4,6,7,11]. Although it is impossible to completely measure and decompose all behavioral and environmental factors into quantitative variables, the positive correlation between cfPWV and years served as a LEO (bivariate correlations and Table 6, Model C) suggests that occupational factors associated with law enforcement may predispose officers to poorer health outcomes, especially the risk of CVD. The increased explained variance from Model C in Table 6 compared to Model A in Table 6 might indicate that time spent serving in law enforcement is a better predictor of cfPWV than just age for this population. However, since years served as a LEO is a function of time, like age, it would be remiss not to report the correlation between the age and LEO years (r = 0.79, *p* < 0.0001).

The lack of significant arterial stiffening in LEOs who participated in third shift for greater than two years was surprising, as previous studies have demonstrated how police shift work (on third shift or late night shift) has created an environment that may promote poor health habits [2,3,4,6,7,11]. This investigation noted that there was a significant positive correlation with time spent on third shift and increased cfPWV. However, no significant difference in cfPWV between LEOs working for more than 2 years on third shift versus LEOs working less than two years on third shift. However, the group mean average cfPWV was up 0.53 m·s^−1^, which is a greater increase compared to Chen et al.’s cross-sectional study which found an increase in pulse wave velocity of 0.036 m·s^−1^ per year of shift work bus driving [36]. Although these populations seem drastically different, they have two major similarities in the fact that they both perform shift work for prolonged periods of time and the both jobs are sedentary in nature [3,37]. Shift work investigations using cfPWV are lacking, but they may help provide a clearer picture of how occupational factors may influence cardiovascular health.

Following the time-based variables of age and years served in law enforcement, this investigation found that relative body fat was positively correlated to arterial stiffness (bivariate correlations, Table 6). That is, as the relative adiposity of a LEO increases, cfPWV also increases. Excess body fat is a well-known accelerant of the arterial stiffening process [20,38]. This investigation agreed with previous research and found that relative body fat (r =0.60, *p* < 0.05) and BMI (r = 0.43, *p* < 0.05) were positively correlated with cfPWV. The stronger association of relative body fat compared to BMI, because BMI was not retained in the regression analyses (Table 6), could be due to the misinterpretation of lean muscle as fat tissue in the BMI equation. Since BMI is solely based on height and weight measures, BMI estimations cannot distinguish between fat and fat-free mass. This evidence supports Alasagheirin’s inquiry calling for a direct measure of fat mass in LEOs instead of BMI, because BMI’s misclassification can add or reduce the risk stratification of health outcomes for officers [39]. This misclassification could hinder prevention timeframes, by delaying early intervention, and thus causing greater harm to an unsuspecting officer.

This investigation demonstrated a significant difference in average cfPWV values based on relative body fat classifications (Table 5). Specifically, the obese group (mean = 7.81 m·s^−1^) had an average cfPWV of nearly 2 m·s^−1^ higher than that of the normal weight group (mean = 5.96 m·s^−1^), despite no difference in the LEO’s age. Thus, a 2 m·s^−1^ greater cfPWV suggests that the obese LEOs have an increased risk of 28% for all cardiovascular events, 30% for cardiovascular mortality, and a 30% increase in all-cause mortality [18]. Increases in arterial stiffness in obese/overweight populations has been attributed to a variety of different factors related to adipokine levels within the body [34,35,38]. Adiponectin is produced by adipose tissue and possesses anti-inflammatory, antiproliferative, antiatherogenic, and insulin-sensitizing mechanisms [38]. Adiponectin has the ability to provide the body with some protective effects, but low levels have been found in obese and hypertensive patients [38]. With limited adiponectin, nitric oxide synthesis expression decreases, which decreases the protective effects of nitric oxide and thus decreases the vessel’s ability to vasodilate. Low levels of adiponectin have been associated with increased arterial stiffness, however the extent of the exact mechanisms are still being investigated [38].

Another adipokine affected by obesity is leptin. Obese individuals have elevated leptin levels in the plasma, which are known to increase aldosterone, sodium retention volume expression, and increased blood pressure [38]. High levels of leptin also contribute to production of vascular smooth muscle cell proliferation, endothelial oxidative stress, and reactive oxidative species formation [38]. There has been an established positive correlation between leptin levels and arterial stiffness [38]. Even though these adipokines may provide a mechanism for increased arterial stiffness, this study did not directly measure these biomarkers and can only speculate as to why there was a significant difference between the three body composition groups.

Hypertension has been established as a correlate of arterial stiffness. Previous research in LEOs has demonstrated that the prevalence of hypertension ranges from 15–39% [5,16]. The present study’s sample was within this range, as 29% of the subjects were classified as hypertensive [40]. For all LEOs, the group average cfPWV (6.72 ± 1.36 m·s^−1^) is within normative range for the group average age and sex (37.1 years, male, cfPWV = 6.50 ± 1.35 m·s^−1^), established by the European Society of Cardiology. Hypertension has a strong link to arterial stiffening, so much so that a few researchers question which occurs first [16]. It has been noted that acute rises in blood pressure can cause acute/chronic increases in arterial stiffness in subjects [15]. These sustained rises in blood pressure may accelerate structural changes within the vasculature walls because hypertensive individuals have elevated stiffness compared to age-matched controls [15]. The fracturing and remolding of connective tissue proteins like elastin and collagen are examples of structural changes within the vascular wall (especially in the ascending aorta) that occur over time due to the increased blood pressure. Even though arterial stiffness has been linked with hypertension, Blacher et al. demonstrated that atherosclerosis alterations, such as arterial wall thickening, calcium build up, and plaque formation, can increase cfPWV independent of age and blood pressure [41]. This would indicate that measuring arterial stiffness in conjuncture with brachial blood pressures would provide health practitioners with a better understanding of the CVD risk of their patients involved in law enforcement.

This investigation evaluated several different multiple regression analyses predicting arterial stiffness as measured by cfPWV and concluded that Model B from Table 6 is the strongest. Model B utilizes age, relative body fat, and brachial diastolic blood pressure to explain 54% (adj. R^2^) of the variance in cfPWV values in this population. The variables of age, body fat, and hypertension have been associated with increases in arterial stiffness in diverse populations [14]. This regression model also provides a simple and practical formula to estimate cfPWV, as a doctor can collect these measures during a yearly physical exam. These simple measures and a normative value table could identify an at-risk LEO earlier, which could lead to primary care interventions to protect LEOs rather than tertiary treatments.

There are several limitations to this study. One major limitation of this study was the voluntary nature of subject recruitment. Many of the officers who were presumed to be in poorer health likely declined to participate in the study. This limitation occurs frequently in research and likely produces a bias in the population’s estimate of health outcome variables. This sentiment is why further testing of LEOs must be completed in conjuncture with administrative support, to truly understand the health disparities that are present in this population. A second limitation was the use of a cross-sectional designed study to assess longitudinal outcomes; however, a cross-section study can still provide important insights for future studies. A third limitation was the recruitment of only males in this study, as it is known that sex-specific traits play a role in cardiovascular health and assessment [42]. Unfortunately, the LEOs in the law enforcement agencies utilized in this study had a sex composition that was approximately ≥90% male. Future research should strive to investigate male and female in law enforcement. A fourth limitation was the use of the Police Stress Questionnaires, as the survey focuses on the last six months and does not give a measure of the full career stress. However, the survey has been investigated to be reliable and valid [24]. A final limitation of this study was the use of currently employed LEOs. These officers are deemed fit for duty, while many officers retire early, because they cannot fulfill the requirements of being a law enforcement officer. This leads to a bias known as the healthy worker effect, which must be investigated in future studies with the law enforcement population.

## 5. Conclusions

In conclusion, the primary findings of this investigation demonstrated that LEOs have significantly lower levels of arterial stiffness earlier in their career, which may be a sign of the healthy worker effect due to the screening process and cadet training requirements prior to full-time employment as a LEO. In contrast, later in the career-span, LEOs demonstrated significantly higher arterial stiffness outcomes compared to the general population. The apparent disproportional increase in arterial stiffness across the career-span suggests that the occupation of law enforcement may contribute to accelerated arterial stiffness. In addition, this investigation confirmed that age and obesity are primary factors in determining arterial stiffness in a cohort of LEOs. The other hypothesized variables showed that time spent on third shift, perceived stress, and moderate-to-vigorous physical activity were not predictors of arterial stiffness in LEOs. Time on third shift and moderate-to-vigorous physical activity did have an interaction effect, which should be further evaluated in future studies. Additionally, years in law enforcement provided strong predictive power of arterial stiffness, which is a novel contributor to arterial stiffness. Occupational longevity as a predictor of arterial stiffness may help inform prospective employees which career choices are at greater risk for CVD; however, this relationship also needs further evaluation. Finally, despite the development of a regression equation to predict cfPWV in LEOs, this equation needs to be cross-validated with an independent sample of LEOs, warranting further research.

Arterial stiffness as measured by cfPWV might be an important tool in diagnosis of CVD risk among LEOs, since many traditional risk factors appear to be absent until post-retirement [6]. Increased arterial stiffness (cfPWV) has been linked to sudden cardiac events independent of traditional CVD risk factors. If direct measurement is unavailable, using a regression model with age, relative body fat, and diastolic blood pressure could provide a reasonable estimation of arterial stiffness among LEOs. This information would be valuable for practitioners and patients by providing an earlier detection of CVD risk and thus identifying candidates for appropriate interventions. This would also provide a benefit to the government and taxpayers through reducing health care costs through using primary or secondary care instead of relying on tertiary care.

In conclusion, older LEOs as well as LEOs who present traditional risk factors such as hypertension and obesity are at greater risk of increased arterial stiffness. This investigation highlights the need for weight and blood pressure management for CVD risk reduction of an at-risk population. The investigation also highlights that age, relative body fat, and diastolic blood pressure are among the strongest predictors of arterial stiffness in this cohort of LEOs.

## Figures and Tables

**Table 1:** Descriptive characteristics of 70 male law enforcement officers.

Variable	Mean	±	SD
Age (years)	37.1	±	7.7
LEO experience (years)	11.0	±	7.6
Height (cm)	179.2	±	6.8
Body mass (kg)	94.5	±	16.4
BMI (kg·m^−2^)	29.4	±	4.5
Waist circumference (cm)	94.3	±	11.1
Abdominal circumference (cm)	97.9	±	12.2
Hip circumference (cm)	104.9	±	8.0
Fat mass (kg)	22.2	±	9.3
Relative body fat (%)	22.8	±	5.6
Fat-free mass (kg)	72.3	±	8.5
Fat-free mass (%)	77.2	±	5.6
Months on first shift	45.5	±	55.8
Months on second shift	47.1	±	43.2
Months on third shift	35.5	±	42.0
PSQ-Op	61.9	±	18.4
PSQ-Org	57.8	±	18.0
PSQ-total	119.7	±	33.7
HEI (*n* = 58)	60.3	±	12.6
Steps per day (*n* = 57)	6977	±	2181
MVPA per day (min) (*n* = 57)	7.4	±	11.0

LEO: law enforcement officer; BMI: body mass index; PSQ-Op: operational police stress questionnaire score; PSQ-Org: organizational police stress questionnaire score; PSQ-total: combined score of operational and organizational police stress questionnaires; HEI: Healthy Eating Index Score; MVPA: moderate-to-vigorous physical activity.

**Table 2:** Resting cardiovascular measurements in 70 male law enforcement officers.

Variable	Mean	±	SD
cfPWV (m·s^−1^)	6.72	±	1.36
Systolic blood pressure (mmHg)	129.77	±	11.15
Diastolic blood pressure (mmHg)	83.84	±	8.00
Pulse pressure (mmHg)	45.93	±	9.00
Aortic systolic pressure (mmHg)	114.54	±	10.21
Aortic diastolic pressure (mmHg)	84.63	±	7.91
Aortic pulse pressure (mmHg)	29.91	±	6.43
Aortic AIx	8.66	±	12.78
Aortic AIx75	0.93	±	12.87
RMSSD	49.62	±	33.02
SDNN	60.60	±	25.43

cfPWV: carotid-femoral pulse wave velocity; Aortic AIx: aortic augmentation index; Aortic AIx75: aortic augmentation index at heart rate 75 beats per minute; RMSSD: root mean squared of successive differences of neighboring R-R intervals; SDNN: Standard deviation of normal-to-normal R-R intervals.

**Table 3:** Comparison of average pulse wave velocity in the general population versus the 70 male law enforcement officers (LEOs) by age strata.

Age (Years)	PWV * (m·s^−1^) Male LEOs (*n* = 70)				PWV * (m·s^−1^) General Population ^†^ (*n* = 1455)			Mean Difference (m·s^−1^)	Rel. Diff. (%)	*p*-Value
<30	5.6 ^a^	±	0.74	(*n* = 15)	6.2	±	0.75	−0.6	−10.2	0.002
30–39	6.6	±	1.13	(*n* = 28)	6.5	±	1.35	0.1	1.5	0.644
40–49	7	±	1.13	(*n* = 22)	7.2	±	1.30	−0.2	−2.8	0.411
50–55	9.4 ^a^	±	0.67	(*n* = 5)	8.3	±	1.90	1.1	12.4	<0.001

* Values represent mean ± standard deviation. ^†^ Normative values adapted from Reference Values for Arterial Stiffness’ Collaboration, 2010. Significance set at *p* < 0.05. ^a^ Significant difference between LEOs and normative values for age strata. PWV: pulse wave velocity; Rel. Diff.: Relative difference between groups calculated as: ((LEO value—General population value)/LEO value) × 100.

**Table 4:** Comparison of descriptive and cardiovascular measures by age strata in 70 male law enforcement officers (mean ± SD).

	<30 years (*n* = 15)			30–39 years (*n* = 28)			40–49 years (*n* = 22)			50–55 years (*n* = 5)		
Age (years) (a, b, c, d, e, f)	27.27	±	1.33	34.57	±	3.19	43.91	±	2.67	51.40	±	1.67
LEO experience (years) (a, b, c, d, e, f)	3.97	±	1.23	8.14	±	4.78	16.77	±	5.61	23.20	±	8.41
BMI (kg·m^−2^)	29.57	±	4.59	28.79	±	4.33	29.43	±	4.50	32.16	±	5.19
Relative body fat (%) (c)	20.71	±	6.07	22.15	±	5.11	23.65	±	4.54	28.86	±	7.03
Waist circumference (cm)	92.46	±	11.29	93.41	±	11.16	94.36	±	10.09	104.98	±	11.73
Systolic blood pressure (mmHg)	130.07	±	11.61	128.64	±	12.22	129.95	±	9.82	134.40	±	10.95
Diastolic blood pressure (mmHg)	81.73	±	7.11	83.89	±	8.83	83.91	±	7.19	89.60	±	8.41
AIx (b, c, d)	0.96	±	11.90	4.54	±	10.72	17.30	±	11.03	16.87	±	8.47
AIx75 (b, c, d)	−8.24	±	11.91	−2.47	±	10.04	9.69	±	11.82	9.00	±	7.05
Aortic systolic pressure (mmHg)	111.89	±	8.11	112.43	±	11.52	117.23	±	9.16	122.47	±	7.56
Aortic diastolic pressure (mmHg)	82.36	±	6.78	84.50	±	9.04	84.91	±	6.50	91.00	±	8.61
RMSSD (*n*: 12, 28, 20, 5)	68.74	±	33.93	49.69	±	35.04	42.50	±	29.45	31.82	±	11.14
SDNN (*n*: 12, 28, 20, 5)	72.51	±	27.44	60.99	±	23.07	56.12	±	28.56	47.80	±	8.82
cfPWV (m·s^−1^) (a, b, c, e, f)	5.60	±	0.74	6.60	±	1.13	7.00	±	1.13	9.40	±	0.67

Significance set at *p* < 0.05. LEO: law enforcement officer; BMI: body mass index; AIx: aortic augmentation index; AIx75: aortic augmentation index at heart rate 75 beats per minute; RMSSD: root mean squared of successive differences; SDNN: standard deviation of normal-to-normal R-R intervals; cfPWV: carotid-femoral pulse wave velocity. a = significant difference between the <30 years group and the 30–39 years group; b = significant difference between the <30 years group and the 40–49 years group; c = significant difference between the <30 years group and the 50–55 years group; d = significant difference between the 30–39 years group and the 40–49 years group; e = significant difference between the 30–39 years group and the 50–55 years group; f = significant difference between the 40–49 years group and the 50–55 years group.

**Table 5:** Comparison of cfPWV according to body composition category in 70 male law enforcement officers, while controlling for age.

Relative Body Fat (%)	*n*	cfPWV (m·s^−1^)			Age		
Obese (>25) (a, b)	21	7.81	±	1.40	39.90	±	7.75
Overweight (21–25) (b)	25	6.54	±	1.13	36.76	±	7.93
Normal (<21) (a)	24	5.96	±	0.91	35.13	±	7.10
Body Mass Index (kg·m^2^)	*n*	cfPWV (m·s^−1^)			Age		
Obese (≥30) (a)	27	7.23	±	1.66	36.96	±	8.96
Overweight (25–29.9)	35	6.54	±	1.04	37.91	±	6.74
Normal (≤24.9) (a)	8	5.82	±	0.85	34.38	±	7.69

Values represent mean ± standard deviation. Significance set at *p* < 0.05. a = a significant difference in cfPWV between obese and normal weight groups for respective assessment of body composition; b: a significant difference in cfPWV between obese and overweight groups for respective assessment of body composition. cfPWV: carotid-femoral pulse wave velocity.

**Table 6:** Multiple linear regression models (A–C) predicting aortic stiffness (cfPWV) with occupational and lifestyle factors in 70 male law enforcement officers.

	A	B	C
Constant	1.2928 *	−1.1834	3.3546 *
	(0.646)	(1.239)	(0.477)
Age	0.0754 **	0.0749 **	
	(0.016)	(0.015)	
%BF	0.1153 **	0.0905 **	0.1088 **
	(0.022)	(0.024)	(0.022)
DBP		0.0365 *	
		(0.016)	
Years LEO			0.0804 **
			(0.016)
R-squared	0.526	0.562	0.539
Adjusted R-squared	0.512	0.542	0.525
No. Observations	70	70	70

* *p* < 0.05, ** *p* < 0.001; significance of model coefficients, (standard error); %BF: relative body fat; DBP: diastolic blood pressure; Years LEO: years as a law enforcement officer; No. Observations: number of observations.

## Data Availability

The data presented in this study are available on request from the corresponding author. The data are not publicly available due to privacy and ethical constraints.

## Supplementary Materials

The following are available online at https://www.mdpi.com/article/10.3390/ijerph181910190/s1, Table S1, Bivariate correlation matrix of carotid-femoral pulse wave velocity verses demographic, occupational, anthropometric, and cardiovascular outcomes in 70 law enforcement officers.
