# Supplementary material for: Predictors of Arterial Stiffness in Law Enforcement Officers
Source: Int J Environ Res Public Health. 2021 Sep 28;18(19):10190. doi: 10.3390/ijerph181910190 (PMC8508055; doi:10.3390/ijerph181910190)
Supplement: Supplementary file 1 [file ijerph-18-10190-s001.zip › ijerph-1356442-supplementary.pdf]

Table S1. Bivariate correlation matrix of carotid-femoral pulse wave velocity verses demographic, occupational, anthropometric, and cardiovascular outcomes in 70 law enforcement officers.

	Age	LEO yrs.	Yrs. 1st Shift	Yrs. 2nd Shift	Yrs. 3rd Shift	PSQ-org	PSQ-op	PSQ-total	BMI	% BF	WC	SBP	DBP	Aortic SBP	Aortic DBP	AIx 75	RMSSD	HEI Total	Steps	MVPA
LEO yrs.	0.79*																			
Yrs. 1st Shift	0.59*	0.68*																		
Yrs. 2nd Shift	0.48*	0.52*	0.12																	
Yrs. 3rd Shift	0.27	0.52*	0.12	-0.11																
PSQ-org	0.19	0.23	0.14	0.12	0.20															
PSQ-op	0.01	-0.01	0.01	-0.08	0.09	0.71*														
PSQ-total	0.11	0.11	0.08	0.02	0.15	0.92*	0.93*													
BMI	0.10	0.19	0.18	0.15	0.10	0.07	0.06	0.07												
% BF	0.30*	0.34*	0.33*	0.19	0.15	0.09	0.06	0.08	0.86*											
WC	0.19	0.31*	0.22	0.20	0.18	0.07	0.07	0.08	0.91*	0.9*										
SBP	0.01	0.23*	0.21*	0.13	0.13	0.26	0.06	0.17	0.55*	0.46*	0.51*									
DBP	0.16	0.43*	0.19*	0.32*	0.25*	-0.02	-0.07	-0.05	0.6*	0.48*	0.64*	0.6*								
Aortic SBP	0.23*	0.47*	0.41*	0.27	0.22	0.19	-0.02	0.09	0.67*	0.61*	0.64*	0.88*	0.76*							
Aortic DBP	0.20*	0.47*	0.23*	0.33*	0.26*	0.02	-0.08	-0.03	0.61*	0.51*	0.66*	0.62*	0.98*	0.78*						
AIx 75	0.54*	0.53*	0.40*	0.35*	0.24	0.02	-0.15	-0.07	0.34*	0.44*	0.32*	0.08	0.32*	0.47*	0.33*					
RMSSD	-0.39*	-0.18	-0.09	-0.15	-0.08	-0.02	0.15	0.07	0.10	0.06	0.06	0.22	0.07	0.16	0.06	-0.14				
HEI Total (n=58)	0.05	0.08	0.01	0.06	0.17	-0.07	-0.11	-0.10	-0.06	-0.21	-0.16	0.01	-0.08	0.00	-0.08	-0.06	-0.13			
Steps (n=57)	0.21	0.20	0.16	-0.01	0.24	-0.05	-0.09	-0.07	-0.27	-0.20	-0.24	-0.22	-0.16	-0.20	-0.10	0.04	-0.19	0.41		
MVPA (n=57)	0.07	0.06	0.01	-0.11	0.08	-0.09	-0.04	-0.07	-0.19	-0.23	-0.25	-0.06	0.00	-0.10	-0.02	-0.10	-0.17	0.26	0.73	
cfPWV	0.57*	0.60*	0.43*	0.32*	0.31*	0.10	-0.02	0.04	0.43*	0.60*	0.53*	0.36*	0.46*	0.54*	0.49*	0.50*	0.54*	-0.11	-0.12	-0.11

*Indicates significant correlation ($p < .05$).

LEO yrs.: years served as Law Enforcement Officer, PSQ-org : Organizational Police Stress Questionnaire score, PSQ-op: Operational Police Stress Questionnaire Score, PSQ-total: Combined score of PSQ-org and PSQ-op, BMI: Body Mass Index, % BF: Relative body fat (%), WC: waist circumference, SBP: brachial systolic blood pressure, DBP: Brachial Diastolic Blood Pressure, Aortic SBP: Aortic Systolic Blood Pressure, Aortic DBP: Aortic Diastolic Blood Pressure, AIx75: aortic augmentation index at heart rate 75 beats per minute, RMSSD: root mean squared of successive differences, HEI Total: Healthy Eating Index Score, Steps: Average steps per day, MVPA: moderate-to-vigorous physical activity, cfPWV: carotid-femoral pulse wave velocity ($m \cdot s^{-1}$).
